# Thrombomodulin protects against lung damage created by high level of oxygen with large tidal volume mechanical ventilation in rats

**DOI:** 10.1186/s40560-014-0057-0

**Published:** 2014-10-01

**Authors:** Yoshiaki Iwashita, Erquan Zhang, Junko Maruyama, Ayumu Yokochi, Yasuharu Yamada, Hirofumi Sawada, Yoshihide Mitani, Hiroshi Imai, Koji Suzuki, Kazuo Maruyama

**Affiliations:** Department of Anesthesiology and Critical Care Medicine, School of Medicine, Mie University, 2-174 Edobashi, Tsu, Mie 5148507 Japan; Department of Pediatrics, School of Medicine, Mie University, Tsu, Mie Japan; Department of Emergency Critical Care Center, Mie University School of Medicine, 2-174 Edobashi, Tsu, Mie 514-8507 Japan; Department of Medical Engineering, Suzuka University of Medical Science, 1001-1 Kishiokacho, Suzuka, Mie 510-0226 Japan; Department of Pharmacological Science, Suzuka University of Medical Science, 1001-1 Kishiokacho, Suzuka, Mie 510-0226 Japan

**Keywords:** Ventilator-induced lung injury, Thrombomodulin, Pulmonary hypertension, Nitric oxide

## Abstract

**Background:**

Ventilator-induced lung injury (VILI) is associated with inflammatory responses in the lung. Thrombomodulin (TM), a component of the coagulation system, has anticoagulant and anti-inflammatory effects. We hypothesized that the administration of recombinant human soluble TM (rhsTM) would block the development of lung injury.

**Methods:**

Lung injury was induced by high tidal volume ventilation for 2 h with 100% oxygen in rats. Rats were ventilated with a tidal volume of 35 ml/kg with pretreatment via a subcutaneous injection of 3 mg/kg rhsTM (HV (high tidal volume)/TM) or saline (HV/SAL) 12 h before mechanical ventilation. Rats ventilated with a tidal volume of 6 ml/kg under 100% oxygen with rhsTM (LV (low tidal volume)/TM) or saline (LV/SAL) were used as controls. Lung protein permeability was determined by Evans blue dye (EBD) extravasation.

**Results:**

Lung injury was successfully induced in the HV/SAL group compared with the LV/SAL group, as shown by the significant decrease in arterial oxygen pressure (PaO_2_), increased protein permeability, and increase in mean pulmonary artery pressure (mPAP) and ratio of mean pulmonary artery pressure to mean artery pressure (Pp/Ps). Treatment of rats with lung injury with rhsTM (HV/TM) significantly attenuated the decrease in PaO_2_ and the increase in both mPAP and Pp/Ps, which was associated with a decrease in the lung protein permeability. Lung tissue mRNA expressions of interleukin (IL)-1α, IL-1β, IL-6, tumor necrosis factor-α, and macrophage inflammatory protein (MIP)-2 were significantly higher in HV than in LV rats. Rats with VILI treated with rhsTM (HV/TM) had significantly lower mRNA expressions of IL-1α, IL-1β, IL-6, and MIP-2 than those expressions in HV/SAL rats.

**Conclusions:**

Administration of rhsTM may prevent the development of lung injury created by high level of oxygen with large tidal volume mechanical ventilation, which has concomitant decrease in proinflammatory cytokine and chemokine expression in the lung.

## Background

Mechanical ventilation can initiate or exacerbate lung injury, causing what is referred to as ventilator-induced lung injury (VILI) [1]. A high transalveolar pressure, tidal volume, and respiratory rate contribute to the development of VILI [2]. Mechanical force not only causes disruption of the lung tissue but also induces biochemical changes in the lung, leading to an upregulation of inflammatory responses, such as increased neutrophil infiltration and inflammatory mediators in bronchoalveolar lavage fluid and the lung tissue [3,4]. Minimizing alveolar overdistension with a low tidal volume attenuates inflammatory changes in VILI [5] and improves the mortality of acute respiratory distress syndrome (ARDS) patients [6] and abdominal surgical patients under general anesthesia [7]. In an animal model of VILI, mechanical ventilation caused a procoagulant and anti-fibrinolytic state, so-called ventilator-associated coagulopathy, as evidenced by an increase in thrombin-antithrombin complex and plasminogen activator inhibitor-1 [8,9].

Thrombomodulin (TM) is a transmembrane glycoprotein receptor for thrombin that has anticoagulant and anti-inflammatory effects [10-12]. Thrombin converts fibrinogen to fibrin. Binding of fibrin to monocytes activates nuclear factor kappa B (NF-κB), which activates proinflammatory cytokine production [9,13]. Thrombin also activates protease-activated receptors (PARs) on the cell surface and subsequently stimulates the production of adhesion molecules in endothelium and leukocytes [14] and interleukin (IL)-6 and IL-8 in endothelial cells [15], thereby potentiating leukocyte chemotaxis and adhesion. These effects of thrombin are associated with VILI, with an earlier study showing an increase in intracellular adhesion molecules in macrophages obtained by the lung lavage from rats with VILI [3].

Binding of TM to thrombin to assemble the thrombin-TM complex has the following effects: (1) activation of protein C to form activated-protein C (APC) which inhibits factors Va and VIIIa and suppresses further thrombin formation; (2) sequestration of thrombin, thereby reducing fibrin formation; (3) formation of thrombin-activatable fibrinolysis inhibitor and subsequent C5a inactivation; and (4) inhibition of high-mobility group box 1 (HMGB-1), a late mediator of sepsis [11,16]. These effects of TM antagonize thrombin’s procoagulant and proinflammatory activity, which are exerted via the TM expressed on vascular endothelial cells [10-12].

Recently, recombinant human soluble TM (rhsTM) was approved for the treatment of disseminated intravascular coagulopathy (DIC) in Japan [12]. Patients with sepsis associated with DIC tend to receive mechanical ventilation under oxygen inhalation at high concentrations. Because we hypothesized that administration of an exogenous TM such as rhsTM would prevent the development of lung injury, the purpose of this study was to determine if rhsTM administration ameliorates lung injury created by high level of oxygen with large-tidal volume mechanical ventilation in rats.

## Methods

### Animals

Male Sprague-Dawley rats (Clea, Japan) weighing 250–350 g were used. The animal experiments committee of the Mie University School of Medicine, Mie, Japan approved the study protocol.

### Preliminary experiment

**Figure 1:**
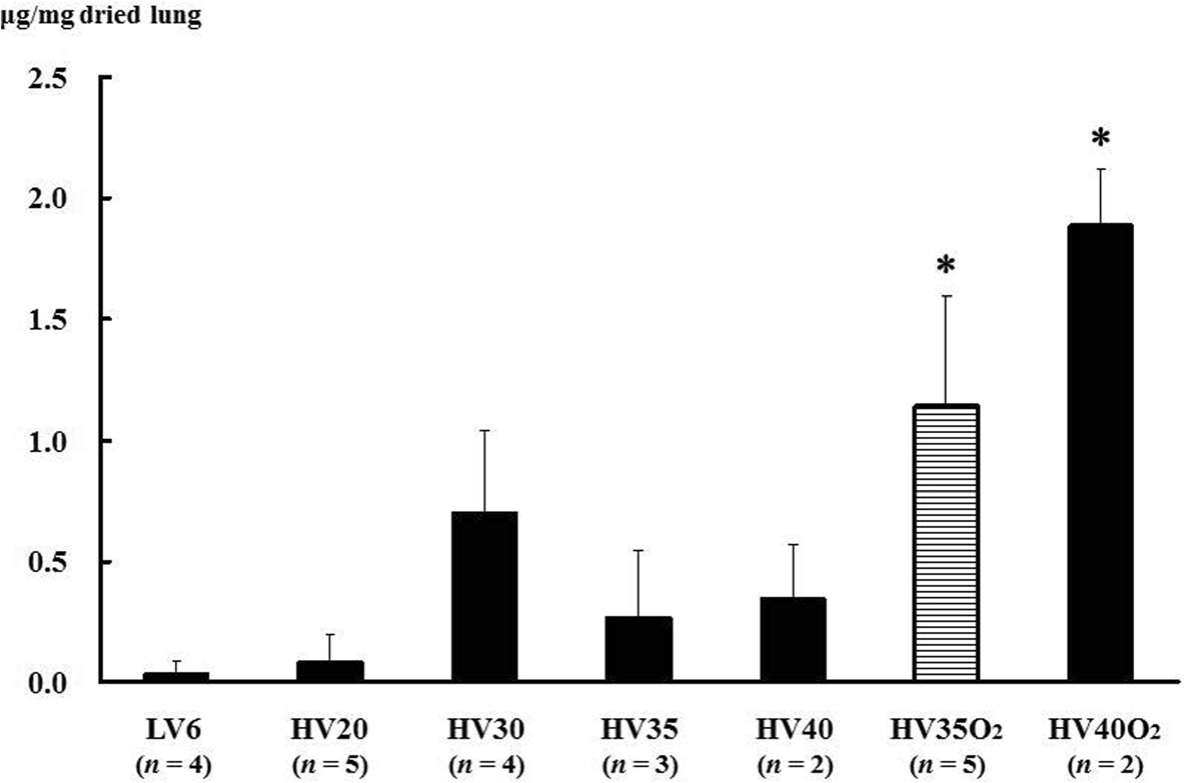
**Preliminary experiment: Evans blue dye (EBD) extravasation as an estimate of lung protein permeability for different tidal volumes, with and without 100% oxygen inhalation.** LV6, rats ventilated under room air with a tidal volume of 6 ml/kg; HV20, HV30, HV35, HV40, rats ventilated under room air with tidal volumes of 20, 30, 35, and 40 ml/kg, respectively; HV35O_2_ and HV40O_2_, rats ventilated with tidal volumes of 35 and 40 ml/kg under 100% oxygen inhalation, respectively. *Bars* indicate mean ± SE. *P < 0.05 versus the LV6 group. *n* = number of rats treated with mechanical ventilation.

In a preliminary experiment performed to select the experimental condition for induction of VILI in rats, 29 rats were assigned to one of the seven groups and ventilated under the following tidal volume conditions: (1) 6 ml/kg under room air (low tidal volume (LV) 6), (2) 20 ml/kg under room air (HV20), (3) 30 ml/kg under room air (HV30), (4) 35 ml/kg under room air (HV35), (5) 40 ml/kg under room air (HV40), (6) 35 ml/kg under 100% oxygen inhalation (HV35O_2_), and (7) 40 ml/kg under 100% oxygen inhalation (HV40O_2_). After mechanical ventilation for 2 h with the assigned tidal volume, Evans blue dye (EBD) extravasation was measured as an index of lung protein permeability. EBD strongly binds to albumin and is used as a marker of protein extravasation in lung injury models [9,17,18]. Based on these results (Figure 1), we decided to induce lung injury via a tidal volume of 35 ml/kg under 100% oxygen inhalation.

### Mechanical ventilation

A tracheostomy was performed under intraperitoneal pentobarbital (45 mg/kg) anesthesia and a plastic cannula (SP-110; Natsume, Tokyo, Japan) was inserted into the trachea. The rats were then ventilated using an SN-480-7 volume cycle ventilator (Shinano Co., Nagoya City, Japan). Muscle relaxation was achieved using pancuronium bromide (0.1 mg/kg). Positive end-expiratory pressure was not applied. To maintain arterial carbon dioxide tension at a pressure between 40–50 mmHg, the respiratory rate was adjusted to be 30–100 breaths/min, and the dead space was adjusted by inserting a tube between the Y-piece of the ventilator circuit and tracheostomy cannula in the high tidal volume group.

### Experimental group

Twenty rats were divided into four groups as follows: (1) rats pretreated with saline and ventilated with a tidal volume of 6 ml/kg under 100% oxygen inhalation (LV/saline (SAL)) (*n* = 5); (2) rats pretreated with rhsTM and ventilated with a tidal volume of 6 ml/kg under 100% oxygen inhalation (LV/TM) (*n* = 5); (3) rats pretreated with saline and ventilated with a tidal volume of 35 ml/kg under 100% oxygen inhalation (HV/SAL) (*n* = 5); and (4) rats pretreated with rhsTM and ventilated with a tidal volume of 35 ml/kg under 100% oxygen inhalation (HV/TM) (*n* = 5). The rats were subcutaneously injected with 3 mg/kg of rhsTM (Asahi Kasei Pharma, Tokyo, Japan) dissolved in saline or saline alone 12 h before the start of the mechanical ventilation. In a previous study, we showed that plasma rhsTM levels were elevated after 9 h and remained elevated for 48 h after subcutaneous rhsTM injection [19].

### Catheterization

The left internal carotid artery was cannulated for measuring artery pressure and sampling for arterial blood gas analysis. A pulmonary artery catheter (Silastic tubing, 0.31 mm inner diameter and 0.64 mm outer diameter) was inserted via the right external jugular vein into the pulmonary artery using the closed-chest technique, as previously described [19,20]. Airway pressure was measured at the tracheostomy cannula. Mean artery pressure (mAP), mean pulmonary artery pressures (mPAP), and airway pressure were recorded with a physiological transducer and amplifier system (AP 620; Nihon Kohden, Tokyo, Japan). The ratio of mPAP to mAP (Pp/Ps) was calculated as an estimate of pulmonary hypertension.

### Experimental protocol

All rats were initially ventilated with a tidal volume of 6 ml/kg. After a 15-min stabilization, an intravenous injection of 30 mg/kg EBD was given. Baseline arterial blood gas analysis was performed before the assignment of experimental tidal volume. After a 30-min stabilization, a tidal volume of 6 or 35 ml/kg was assigned, and mechanical ventilation was continued for another 2 h. Further arterial blood samples were obtained every 30 min after the assignment of a tidal volume and were analyzed by a portable blood gas analyzer (iSTAT Analyzer 200; Abbott Point Care Inc., Princeton, NJ, USA). Alveolar-arterial oxygen difference (A-aDO_2_) was calculated with the following formula: A-aDO_2_ = (760 − 47) × 1.0 − PaCO_2_/0.8 − PaO2 = 713 – PaCO_2_/0.8 − PaO2. At the end of the protocol, the lung tissue was obtained to measure the lung water content and pulmonary microvascular permeability.

### EBD extravasation and the lung dry/wet weight ratio

EBD extravasation into the lung as an estimate of protein permeability was quantitated as previously described [17,18]. After thoracotomy, the upper lobe of the right lung was ligated and harvested to determine the lung water content. The rest of the lung was perfused via the pulmonary artery for 2 min with saline to eliminate residual blood and EBD from the pulmonary bed [18]. After perfusion, the left lung was excised, rinsed externally with saline, and then placed in a drying oven at 90°C for 24 h. Dried tissue samples were placed in 2 ml formamide at 37°C for 24 h. The EBD concentration was measured by dual wavelength spectrophotometry at 620 and 740 nm, which allows for the correction of contaminating heme pigments. The corrected absorbance at 620 nm was calculated using the following formula: Corrected absorbance at 620 mm = actual absorbance at 620 mm − [1.426 (absorbance at 740 mm) − 0.03]. The total amount of EBD in the dried lung sample was calculated, and EBD extravasation was expressed as micrograms per gram of the dried lung tissue sample. The upper lobe of the right lung was placed in a drying oven at 90°C for 24 h. The lung dry-to-wet weight (dry/wet) ratio as an estimate of lung water content was determined by the following formula: (wet lung weight – dried lung weight)/wet lung weight × 100 (%).

### cDNA preparation and real-time polymerase chain reaction

In another set of experiments with 21 rats [(LV/SAL) (*n* = 5), LV/TM (*n* = 5), HV/SAL (*n* = 6), and HV/TM (*n* = 5)], the lung samples were obtained for real-time polymerase chain reaction (PCR) and Western blotting. Interleukin (IL)-1α, IL-1β, IL-6, tumor necrosis factor-α (TNFα), macrophage inflammatory protein (MIP)-2, rho-associated kinase (ROCK)-1, and HMGB-1 mRNA levels were determined by real-time PCR. After the extraction of total RNA from whole lung tissue [LV/SAL (*n* = 5), LV/TM (*n* = 5), HV/SAL (*n* = 6), HV/TM (*n* = 5)] using TRIzol reagent (Invitrogen, Carlsbad, CA, USA), cDNA synthesis was performed with ReverTra Ace (Toyobo Co., Ltd., Biochemical Operations Department, Osaka, Japan). Amplification was performed with a StepOne Plus Real Time PCR System (Applied Biosystems, Thermo Fisher Scientific, Waltham, MA, USA). The sequences of the primer pairs are listed in Table 1. Relative quantification was performed with the comparative ∆∆Ct method by normalization with β-actin mRNA.

**Table 1:** **Primer list**

**Gene name**	**Primer (5′-3′) sequence**
IL-1α	F: AAGACAAGCCTGTGTTGCTGAAGG
	R: TCCCAGAAGAAAATGAGGTCGGTC
IL-1β	F: CACCTCTCAAGCAGAGCACAG
	R: GGGTTCCATGGTGAAGTCAAC
IL-6	F: TCCTACCCCAACTTCCAATGCTC
	R: TTGGATGGTCTTGGTCCTTAGCC
MIP-2	F: CCAACCATCAGGGTACAGGG
	R: GGGTCGTCAGGCATTGACA
HMGB-1	F: GGCTGACAAGGCTCGTTATG
	R GGGCGGTACTCAGAACAGAA
TNFα	F: AAATGGGCTCCCTCTCATCAGTTC
	R: TCTGCTTGGTGGTTTGCTACGAC
ROCK-1	F: TCTCATTTGTGCCTTCCTTACG
	R: GTTTCCCAAGCCCACTGATC
ACTB	F: GACGGTCAGGTCATCACTATCG
	R: TAG TTTCATGGATGCCACAGGAT

### Western blotting

Western blotting for ROCK-1 and HMGB-1 in whole lung tissue was performed as described previously [19]. Three kinds of primary antibodies (anti-HMGB-1, 1:5,000, #3935S, (Cell Signaling Technology, Danvers, MA, USA); anti-ROCK-1, 1:5,000 dilution, 611136, (BD Transduction Laboratories, San Jose, CA, USA); anti-β-actin, 1:200,000 dilution, A5441 (Sigma-Aldrich, St. Louis, MO, USA)) were incubated at 4°C overnight. Secondary antibodies (anti-mouse IgG-HRP, 1:20,000 dilution, NA 931, Amersham; anti-rabbit IgG-HRP, 1:20,000 dilution, D2313, Santa Cruz Biotechnology, Dallas, TX, USA) were incubated for 1 h at room temperature.

### Data analysis

Values are expressed as mean ± SE. When more than two means were compared, one-way analysis of variance was used. When significant variance was found, Fisher’s predicted least significant difference test was used to establish which groups were different. *P* < 0.05 was considered to be significant.

## Results

### Preliminary experiment: effect of tidal volume and 100% oxygen inhalation

In a preliminary experiment, we evaluated the conditions required to induce experimental lung injury in rats. EBD extravasation increased with escalation in tidal volume but did not reach significant difference in room air, probably because of the relatively small number of samples. Under 100% O_2_ inhalation, EBD extravasation was significantly increased in the HV35O_2_ and HV40O_2_ groups compared with the LV6 group (Figure 1). There was no significant difference between the LV6 group and the HV20, HV30, HV35, or HV40 groups. Overall, the results suggested that a high tidal volume with 100% oxygen tended to cause high EBD extravasation, an estimate of the lung protein permeability. We wanted to select the specific condition for obtaining a stable lung injury model in an experimental setting. Two out of four rats in the HV40 and HV40O_2_ groups became unstable and died so we decided to induce lung injury in rats by ventilating with 35 ml/kg and 100% oxygen in subsequent experiments in which the protective effects of TM for the development of VILI were determined. Several other studies also induced lung injury under 100% oxygen inhalation [2,21,22]. An earlier study showed that ventilation with 35 ml/kg tidal volume for 156 min compromise systemic circulation, leading to shock [23], in which ventilation time is longer than our study (2 h).

### Arterial oxygen pressure, A-aDO_2,_ EBD extravasation, and the lung dry/wet weight ratio

**Figure 2:**
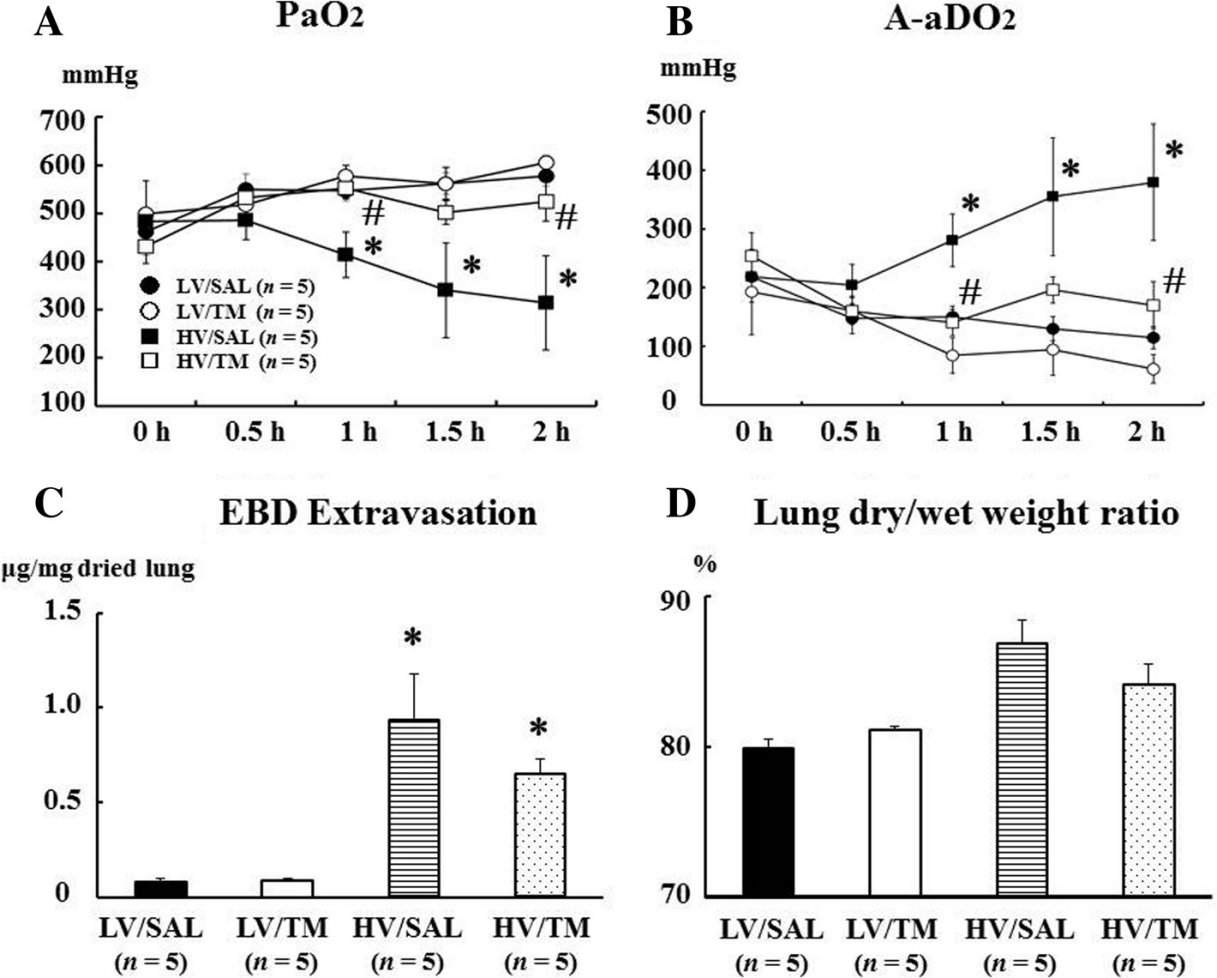
**Arterial oxygen pressure, A-aDO**_**2**_ **, Evans blue dye (EBD) extravasation, and the lung dry/wet weight ratio. (A)** Arterial oxygen pressure (PaO_2_), **(B)** alveolar-arterial oxygen difference (A-aDO_2_), **(C)** EBD extravasation, an estimate of protein permeability, **(D)** the lung dry-to-wet (dry/wet) weight ratio, an estimate of the lung water content. *LV* low tidal volume (6 ml/kg), *HV* high tidal volume (35 ml/kg), *SAL* saline, *TM* thrombomodulin. 0 h, baseline measurement (tidal volume of 6 ml/kg) before the assignment of experimental tidal volume; 0.5, 1, 1.5, and 2 h, hours after the assignment of experimental tidal volume (6 ml/kg or 35 ml/kg). *Bars* indicate mean ± SE. *P < 0.05 versus the respective LV groups. #*P* < 0.05 versus the HV/SAL group. *n* = number of rats.

PaO_2_ was significantly decreased in the HV/SAL group compared with the LV/SAL group at 1, 1.5, and 2 h after the start of mechanical ventilation. The decrease in PaO_2_ was significantly attenuated in the HV/TM group compared with the HV/SAL group at 1 and 2 h. There was no difference in PaO_2_ between the HV/TM and LV groups. Thus, the decrease in PaO_2_ with high tidal ventilation was prevented by TM treatment (Figure 2A). These results were confirmed with A-aDO_2_, an estimate of oxygenation that excludes the effect of alveolar carbon dioxide. EBD extravasation, a marker of protein permeability, was significantly increased in the HV/SAL group and HV/TM group compared with the LV/SAL group and LV/TM group, respectively (Figure 2C). Although not significant, the lung dry/wet ratio tended to be increased in the HV/SAL group and HV/TM group compared with the LV/SAL group and LV/TM group, respectively (Figure 2D). The increase in EBD extravasation and the lung dry/wet weight ratio was lower in HV/TM rats than in HV/SAL rats (Figure 2C, D) but did not reach significance because of small number of experiment animals. Overall, these results showed that the HV/TM group had less lung injury, as measured by three independent methods, than the HV/SAL (positive control) group, suggesting that TM administration might ameliorate lung injury associated with high tidal volume ventilation.

### mPAP and Pp/Ps

**Figure 3:**
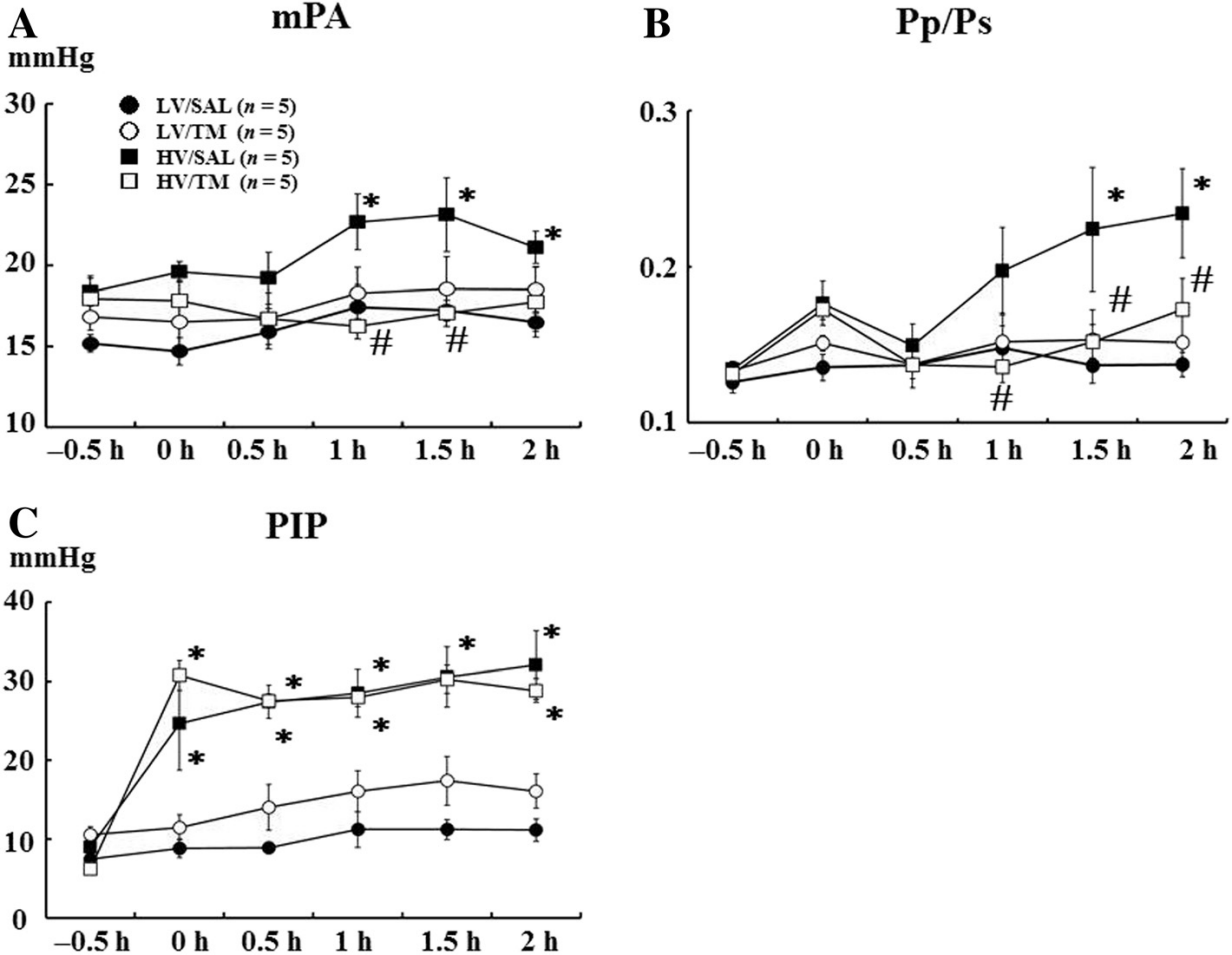
**Mean pulmonary artery pressure, Pp/Ps, and peak inspiratory airway pressure. (A)** Mean pulmonary artery pressure (mPAP), **(B)** the ratio of mean pulmonary artery pressure to mean artery pressure (Pp/Ps), **(C)** peak inspiratory airway pressure (PIP). **(B)** −0.5 h, baseline measurement before the start of experimental tidal volumes. See Figure 2 for other abbreviations. *Bars* indicate mean ± SE. *P < 0.05 versus the respective LV groups. #*P* < 0.05 versus the HV/SAL group. *n* = number of rats.

Because pulmonary artery pressure increases in severe lung injury [24], we measured mPAP. As we expected, mPAP was significantly higher in HV/SAL rats than in LV/SAL rats at 1, 1.5, and 2 h after the start of mechanical ventilation. The increase in mPAP was significantly attenuated in the HV/TM group compared with the HV/SAL group at 1 and 1.5 h. There were no differences in the mPAP between the HV/TM and LV groups (Figure 3A), suggesting that TM treatment prevented the increase in mPAP caused by high tidal volume ventilation. Similar results were obtained for Pp/Ps, an index of pulmonary hypertension [25]. Pp/Ps was significantly higher in HV/SAL rats than LV/SAL rats at 1.5 and 2 h after the start of mechanical ventilation. The increase in Pp/Ps was significantly attenuated in the HV/TM group compared with the HV/SAL group at 1, 1.5, and 2 h. There were no differences in the Pp/Ps between the HV/TM group and LV groups (Figure 3B). The results of mPAP and Pp/Ps are suggestive of the effects of TM-inhibiting pulmonary hypertension induced by hypercytokinemia due to the lung injury created by high level of oxygen with large-tidal volume mechanical ventilation.

### Peak inspiratory airway pressure

Peak inspiratory airway pressure (PIP) was significantly increased in the HV/SAL group and HV/TM group compared with the LV/SAL group and LV/TM group, respectively. There was no difference between the HV/TM group and HV/SAL group (Figure 3C). PIP might be insensitive to detect the effect of TM on the lung injury created by high level of oxygen with large-tidal volume mechanical ventilation.

### Inflammatory cytokines and chemokines

**Figure 4:**
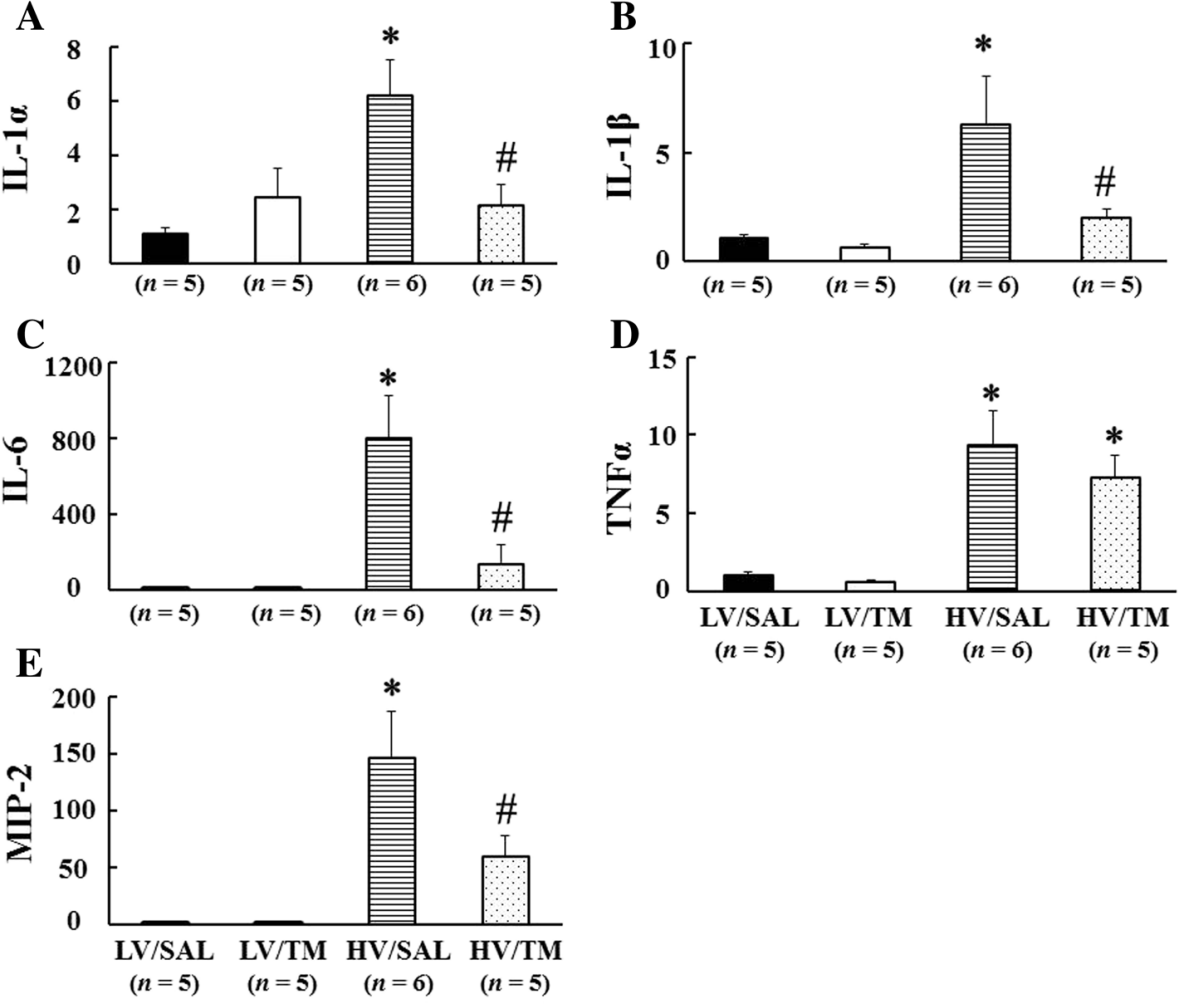
**rhsTM attenuated the increase in cytokine and chemokine mRNA in VILI in rats.** Cytokine [interleukin (IL)-1α **(A)**, IL-1β **(B)**, IL-6 **(C)**, tumor necrosis factor α (TNFα) **(D)**] and chemokine (macrophage inflammatory protein (MIP)-2) **(E)** mRNA expressions in the lung tissue were significantly higher in the HV/SAL group than in the LV/SAL group. Note significant differences between the HV/TM group and HV/SAL group for IL-1α, IL-1β, IL-6, and MIP-2. *P < 0.05 versus the LV group. #*P* < 0.05 versus the HV/SAL group. *n* = number of rats. See Figure 2 for abbreviations.

To confirm that the inflammatory response is accentuated in HV/SAL rats compared with LV/SAL rats, we measured cytokine and chemokine mRNA levels in the lungs. IL-1α, IL-1β, IL-6, TNFα, and MIP-2 mRNA levels in the lungs were significantly higher in the HV/SAL group than in the LV/SAL group. These increases in IL-1α, IL-1β, IL-6, and MIP-2 mRNA levels were significantly attenuated in the HV/TM group compared with the HV/SAL group (Figure 4). There was no difference in the level of TNFα mRNA between the HV/TM group and HV/SAL group.

### ROCK-1

**Figure 5:**
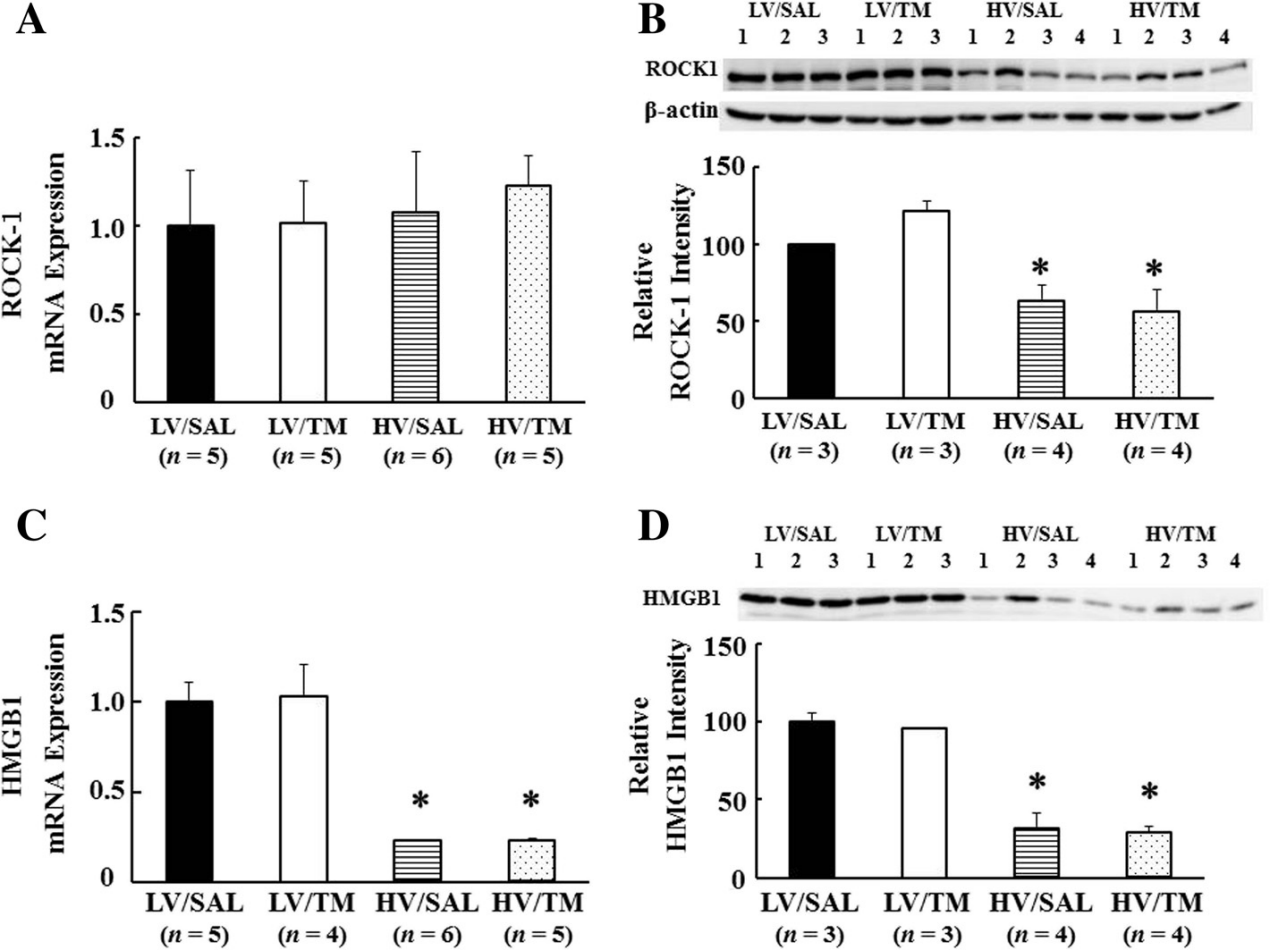
**ROCK-1 and HMGB-1. (A)** Rho-associated kinase (ROCK)-1 mRNA, **(B)** ROCK-1 protein expression, **(C)** high-mobility group box 1 (HMGB-1) mRNA, **(D)** HMGB-1 protein expression. For protein expression, the average intensity of LV/SAL was taken as 100%. Each sample intensity as a percentage of the average was calculated (relative intensity). *P < 0.05 versus the respective LV group. *n* = number of rats. See Figure 2 for abbreviations.

Because ROCK-1 protein level increased in the lung tissue in mice VILI model [9], we determined ROCK-1 mRNA and protein level in the lung tissue with and without TM treatment. There was no change in ROCK-1 mRNA expression among the groups (Figure 5A). ROCK-1 protein expression was significantly decreased in the HV/SAL group and HV/TM group compared with the LV/SAL group and LV/TM group, respectively (Figure 5B). No difference was found between the HV/SAL and HV/TM groups.

### HMGB-1

Because TM has been shown to neutralize HMGB-1, a mediator of inflammation and increased permeability through activation of inducible nitric oxide synthase [16,26], we investigated whether TM administration would reduce HMGB-1 mRNA and protein expressions in the lung in high tidal ventilation. Unexpectedly, HMGB-1 mRNA and protein expressions in the lung were significantly decreased in the HV/SAL group and HV/TM group compared with the LV/SAL group and LV/TM group, respectively. No difference was found between the HV/SAL and HV/TM groups (Figure 5C).

## Discussion

The results of this study showed that high tidal volume ventilation with 100% oxygen induced lung injury, associated with the upregulation of proinflammatory cytokine mRNA in the injured lung. TM pretreatment attenuated the decrease in PaO_2_, lung permeability, and mPAP in VILI rats. Proinflammatory cytokine mRNA levels in the injured lung were decreased in VILI rats treated with TM.

Ventilation with high tidal volume and 100% oxygen significantly upregulated protein permeability measured by EBD extravasation into the lung in rats. The lung dry/wet weight ratio was also nonsignificantly increased in the HV/SAL group compared with the LV/SAL group. These results suggest that EBD extravasation is more sensitive for detecting the increase in protein permeability in the lung than dry/wet weight ratios in the early phase of VILI. The EBD extravasation of LV with room air (LV group in preliminary experiment) and LV with 100% oxygen (LV/SAL group in main experiment) were similar. Thus, we could not detect apparent oxygen toxicity in the present experimental condition. These results indicate our lung injury model was induced by combined high level of oxygen and large tidal volume mechanical ventilation.

The results showed that TM administration prevented VILI, as evidenced by the reduction in protein leakage, attenuation of the decrease in arterial oxygenation in high tidal volume ventilation, and prevention of the increase in mPAP and Pp/Ps ratio. Although this protective effect of rhsTM was associated with a reduction of chemokine (MIP-2) and cytokine (IL-1α, IL-1β, and IL-6) mRNA expression in the lungs with high volume ventilation, a causal relationship between VILI and these mediators remains to be determined [1]. An earlier study showed that IL-1 receptor antagonist protects against VILI in rabbits, suggesting that IL-1 is a candidate mediator of VILI [27].

### Cytokines and chemokines

Expressions of proinflammatory cytokines (IL-1α, IL-1β, IL-6, and TNFα) in TM-untreated lung were significantly elevated following high volume ventilation, which confirms that a high volume ventilation is associated with a proinflammatory reaction. Since TNFα and IL-1β suppress TM mRNA in endothelial cells [11,28], the increase in these cytokines in the present study shows a possible lack of TM during high volume ventilation. A lack of TM is associated with activation of mitogen-activated kinase and an increase in NFκB and ICAM expression [11], thereby potentiating the inflammatory reaction. If there is a lack of TM during high volume ventilation, TM administration could be used as a supplementation therapy. Because TM administration attenuates the increase in IL-6 and IL-1β mRNA, the inflammatory response might be attenuated by TM supplementation. Additional experiment such as determination of TM concentration or TM staining in the lung tissue is necessary.

ROCK-1 protein expression was reduced in rats with high volume tidal ventilation, and TM had no effect on ROCK-1 expression. A previous study that found that expression of ROCK-1 in the lung was increased in VILI mice showed that APC-mediated vascular protection involves inhibition of ROCK-1 [9]. Upregulation of ROCK-1 increases vascular permeability through endothelial gap formation [29]. However, this mechanism was not the case in the present study because ROCK-1 mRNA expression was unchanged. Species difference and model difference might explain the difference of ROCK-1 expression. Furthermore, TM administration might not increase APC in the present study, because rhsTM activates protein C in human and monkey plasma but not in rat and rabbit [30].

Lung tissue MIP-2 mRNA levels were significantly reduced by TM treatment, which might delay the occurrence of early VILI. MIP-2, a rodent homologue of human IL-8 [31], is a potent monocyte attractant, initiating cellular migration and activation and inflammatory cell recruitment.

Since TM binds to HMGB-1 and promotes its degradation and inactivation in sepsis [12,16,26], we measured HMGB-1 mRNA and protein expressions in the lung. Unexpectedly, HMGB-1 mRNA and protein expressions in the lungs were reduced in rats ventilated with high tidal volume. The decrease in HMGB-1 protein possibly might be due to less production since the HMGB-1 mRNA level was downregulated in rats with lung injury created by high level of oxygen with large tidal volume mechanical ventilation. Another possibility is that HMGB-1 protein might be released into the systemic circulation from the lungs during the 2-h ventilation. HMGB-1 is normally present as a nuclear protein [32] and is passively released from damaged cells [26]. A recent study showed that the increase in serum HMGB-1 is associated with the concurrent decrease in tissue HMGB-1 protein expression of the liver in rat sinusoidal obstruction syndrome [33]. Since macrophages produce HMGB-1, the decrease in the number of macrophages might also explain the decrease in HMGB-1 protein levels in the lung tissue. An earlier study of bronchoalveolar lavage fluid and histology demonstrated that alveolar macrophages decreased in the VILI model [3]. We could not detect the effect of TM on HMGB-1 levels in the lung tissue. However, this result does not eliminate the possibility that TM binds plasma HMGB-1 that has been spread from the lung.

### Limitations and clinical implications

The first limitation of this study is that it is observational and not mechanistic. Although observational, this is the first report showing that TM blocks the development of lung injury created by high level of oxygen with large tidal volume mechanical ventilation in an animal model. rhsTM (Asahi Kasei Pharma, Tokyo, Japan) has been approved in Japan since 2008 for the treatment of DIC with hematologic malignancy or infection. Following a phase IIb global trial [32], a subsequent phase III global trial of rhsTM for patients with sepsis-associated coagulopathy is now underway. An observational clinical study showed improvement in respiratory dysfunction in patients with severe sepsis, in which the therapies included mechanical ventilation [34]. Thus, the numbers of septic patients under both mechanical ventilation and rhsTM administration have been increasing [35]. If rhsTM blocks the development of VILI, this beneficial effect would not be limited to just those patients with coagulopathy. Second, we could not differentiate between the effect of high tidal volume and 100% oxygen. The model of VILI should be clearly different from oxygen toxicity, although the latter exacerbates the pathology of the former. Since our preliminary study showed difficulties to induce VILI with high tidal volume ventilation (30–40 ml/kg) in room air and VILI was induced with 100% oxygen inhalation in other studies [2,21-23], our rats were ventilated with high tidal volume with 100% oxygen inhalation. Two hit lung injury, i.e., high tidal volume and 100% oxygen inhalation, induced lung injury in the present study. In patients under mechanical ventilation, high oxygen inhalation is relatively common. Third, in the present study, healthy lungs were ventilated with a tidal volume of 35 ml/kg with resultant high peak inspiratory pressure. Although a tidal volume of 35 ml/kg is unrealistic in the clinical setting, a moderate tidal volume of 10–12 ml/kg in patients with ARDS might cause a region of overdistension equivalent to that of 40 ml/kg ventilation because of nonhomogeneity due to a mixture of ventilated and nonventilated regions in the lung [1]. Fourth, the duration of mechanical ventilation was short, as in other similar studies [1,3,4,9], which is different from the clinical setting. Thus, we can only say that even short-term high tidal ventilation can induce VILI. Fifth, the number of animals might be small to judge the exact effect of TM on VILI. From the ethical standpoint of animal experiment, we saved the number of animals to be sacrificed.

## Conclusions

In summary, the results of this study show that rhsTM administration protects against the development of lung injury in an experimental setting, which has concomitant decrease in cytokine mRNA expression in the lung tissue from rats with the lung injury created by high level of oxygen with large tidal volume mechanical ventilation.
